# Photodynamic Therapy Targeting Macrophages Using IRDye700DX-Liposomes Decreases Experimental Arthritis Development

**DOI:** 10.3390/pharmaceutics13111868

**Published:** 2021-11-05

**Authors:** Daphne N. Dorst, Marti Boss, Mark Rijpkema, Birgitte Walgreen, Monique M. A. Helsen, Desirée L. Bos, Louis van Bloois, Gerrit Storm, Maarten Brom, Peter Laverman, Peter M. van der Kraan, Mijke Buitinga, Marije I. Koenders, Martin Gotthardt

**Affiliations:** 1 Department of Medical Imaging, Radboudumc, Radboud University, 6525 XZ Nijmegen, The Netherlands; marti.boss@radboudumc.nl (M.B.); Mark.Rijpkema@radboudumc.nl (M.R.); desiree.bos@radboudumc.nl (D.L.B.); maarten.brom@radboudumc.nl (M.B.); peter.laverman@radboudumc.nl (P.L.); martin.gotthardt@radboudumc.nl (M.G.); 2 Department of Experimental Rheumatology, Radboudumc, Radboud University, 6525 XZ Nijmegen, The Netherlands; birgitte.walgreen@radboudumc.nl (B.W.); monique.helsen@radboudumc.nl (M.M.A.H.); Peter.vanderKraan@radboudumc.nl (P.M.v.d.K.); marije.koenders@radboudumc.nl (M.I.K.); 3 Department of Pharmaceutics, Utrecht Institute for Pharmaceutical Sciences, Utrecht University, 3508 TC Utrecht, The Netherlands; L.vanBloois@uu.nl (L.v.B.); G.Storm@uu.nl (G.S.); 4 Department of Biomaterials Science and Technology, University of Twente, 7522 NL Enschede, The Netherlands; 5 Department of Nutrition and Movement Science, Maastricht University, 6229 HX Maastricht, The Netherlands; m.buitinga@maastrichtuniversity.nl; 6 Department of Radiology and Nuclear Medicine, Maastricht University, 6229 HX Maastricht, The Netherlands

**Keywords:** photodynamic therapy, liposomes, experimental arthritis

## Abstract

Macrophages play a crucial role in the initiation and progression of rheumatoid arthritis (RA). Liposomes can be used to deliver therapeutics to macrophages by exploiting their phagocytic ability. However, since macrophages serve as the immune system’s first responders, it is inadvisable to systemically deplete these cells. By loading the liposomes with the photosensitizer IRDye700DX, we have developed and tested a novel way to perform photodynamic therapy (PDT) on macrophages in inflamed joints. PEGylated liposomes were created using the film method and post-inserted with micelles containing IRDye700DX. For radiolabeling, a chelator was also incorporated. RAW 264.7 cells were incubated with liposomes with or without IRDye700DX and exposed to 689 nm light. Viability was determined using CellTiterGlo. Subsequently, biodistribution and PDT studies were performed on mice with collagen-induced arthritis (CIA). PDT using IRDye700DX-loaded liposomes efficiently induced cell death in vitro, whilst no cell death was observed using the control liposomes. Biodistribution of the two compounds in CIA mice was comparable with excellent correlation of the uptake with macroscopic and microscopic arthritis scores. Treatment with 700DX-loaded liposomes significantly delayed arthritis development. Here we have shown the proof-of-principle of performing PDT in arthritic joints using IRDye700DX-loaded liposomes, allowing locoregional treatment of arthritis.

## 1. Introduction

Rheumatoid arthritis (RA) is a chronic autoimmune disease affecting the synovial joints in 0.5–1% of the general population [1]. The disease is characterized by relapsing and remitting inflammation associated with progressive damage to the joints [2]. Treatment of RA consists of disease-modifying anti-rheumatic drugs (DMARDs). Despite the treatment paradigm having changed, a considerable percentage of patients do not respond adequately to both biological and synthetic DMARD treatment [3].

In RA, the synovial lining, a thin layer of cells providing a barrier between the synovial fluid and the joint tissue, becomes hyperproliferative, infiltrated with immune cells, and forms a tumor-like pannus. A key cell already present in this resident synovial lining is the macrophage. Its phagocytic function is crucial for maintaining joint homeostasis [4]. In RA, the function of the macrophage changes as it adapts a pro-inflammatory phenotype. Through its secretion of pro-inflammatory factors, such as tumor necrosis factor-α (TNF-α), interleukin (IL) −1, −6, −10 and granulocyte-macrophage colony-stimulating factor, it actively contributes to chemotaxis and activation of infiltrating immune cells [5,6]. Furthermore, these cytokines stimulate resident synovial fibroblasts to adapt and propagate an activated and destructive phenotype [6]. Through their production of enzymes such as matrix metalloproteinases, macrophages also directly contribute to joint destruction [6,7]. Due to the crucial role of macrophages in the initiation and propagation of arthritis, they are an interesting therapeutic target. Liposomes can be used to target macrophages since they are preferentially phagocytosed by these cells [8,9,10,11]. Depleting macrophages with clodronate-loaded liposomes decreased arthritis in rodents [12,13,14] and patients [15]. However, systemic administration of clodronate liposomes also resulted in depletion of these cells from the spleen and liver [14]. This may have serious side effects, such as a higher susceptibility to infection and cancer, and is therefore not an advisable strategy.

Instead, the selective depletion of macrophages only in the affected synovial joint would be a preferred treatment method. Therefore, loading the liposomes with a drug that is constantly active, such as clodronate, and that will thus exert its function in all cells that phagocytose it, is not optimal. Instead, a preferable approach would be to load the liposomes used for macrophage targeting with an inert molecule that can selectively be activated at the sites of inflammation. To this end, photodynamic therapy (PDT) may be a promising therapeutic approach. In PDT, a light-sensitive molecule, a so-called photosensitizer (PS), is used. By exposing the region to be treated to light of a specific wavelength, the PS will produce reactive oxygen species (ROS), which are cytotoxic to the cells in its vicinity. Off-target effects are limited, since the photosensitizer only becomes active upon excitation by locally applied light of a PS-specific wave-length (690 nm for the IRDye700DX used in this study), thus also avoiding side effects by daylight exposure. IRDye700DX (Pubchem ID: 102004325) is a phthalocyanine dye with two siloxane chains to improve water solubility. A NHS ester can be attached to the photosensitizer, which facilitates conjugation to free amino groups on macromolecules. This has previously been used by our group to modulate arthritis when conjugated to a monoclonal antibody targeting fibroblast activation protein [16].

Here, we investigate if targeting the macrophages using liposomes loaded with the PS IRDye700DX (700DX-liposomes) can specifically kill macrophages in vitro as well as decrease arthritis severity in the collagen-induced arthritis (CIA) mouse model.

## 2. Materials and Methods

### 2.1. Animals

Male DBA/1JRj mice were purchased from Janvier-Elevage at 10–12 weeks of age. Mice were housed in conventional cages on a 12 h day–night cycle and fed standard chow ad libitum. The study protocol was approved by the Radboud University animal ethics committee (RU-DEC-2016-0076, CCD number AVD103002016786). All procedures were performed according to the Institute of Laboratory Animal Research guide for Laboratory Animals.

### 2.2. Liposome Preparation and Characterization

Liposomes were prepared based on the film method [17]. Briefly, dipalmitoyl phosphatidylcholine (DPPC) and PEG-(2000)-distearoyl phosphatidylethanolamine (PEG-(2000)-DSPE), obtained from Lipoid AG (Steinhausen, Switzerland); DSPE-DTPA, from Avanti Polar Lipids (Birmingham, UK); cholesterol, obtained from Sigma (St. Louis, MO, USA), were used. All other chemicals were of reagent grade. A mix of chloroform and methanol (10:1 *v/v*) containing DPPC, PEG-(2000)-DSPE, cholesterol and DSPE-DTPA was prepared in a molar ratio of 1.85:0.15:1:0.15. The organic phase was evaporated with a rotavapor (BUCHI Labortechnik AG, Flawil, Switzerland), followed by nitrogen flushing to remove residual organic solvent. Liposomes were dispersed in phosphate-buffered saline (PBS). The liposomes were sequentially extruded through two stacked polycarbonate filters with pore sizes of 600, 200, and 100 nm (Nuclepore, Pleaston, CA, USA) under nitrogen pressure, using a Lipex high pressure extruder (Lipex, Nortern Lipids, Vancouver, BC, Canada). The retrieved liposomes had a mean particle size of 120 nm with a PDI (polydispersity index) of 0.02.

700DX-liposomes were prepared by post-insertion of micelles as described previously [18,19]. Briefly, micelles were prepared by mixing DSPE-NH_2_-PEG-2000 and DSPE-PEG-2000 (both Avanti Polar Lipids, Birmingham, UK) in a 1:1 molar ratio to enable covalent coupling of IRDye-700DX NHS ester (LI-COR Biosciences, Lincoln, NE, USA) to the NH_2_ terminus of the PEG conjugates. Subsequently, micelles were incubated with the liposomes at 60 °C to allow post-insertion of the PEG conjugates. Unbound IRDye700DX was removed from solution using using a Slide-A-Lyzer Dialysis Cassette (20.000 MWCO, Thermofisher, Waltham, MA, USA) in phosphate-buffered saline (PBS) with 0.5% *w/v* Chelex (Bio-Rad, Hercules, CA, USA).

Fluorescence and absorbance spectra of the liposomes with and without IRDye700DX, as well as those of the free PS, were compared using a TECAN infinite M200 Pro plate reader (PerkinElmer, Groningen, The Netherlands). For fluorescence emission spectrum measurements, an excitation wavelength of 650 nm was used. Results were normalized to the peak absorbance for comparison of the different compounds.

### 2.3. In Vitro Photodynamic Therapy

RAW 264.7 cells were cultured in Dulbecco’s modified Eagle’s medium (DMEM) with 4.5 g/L D-glucose and Glutamax, supplemented with 10% fetal calf serum (FCS), 100 IU/mL penicillin G and 10mg/mL streptomycin. Cells were seeded into 24-well plates (Thermo Scientific, Waltham, MA, USA) (150,000 cells/well) and grown overnight (*n* = 3 wells/group). Medium was replaced by binding buffer (medium with 0.1% bovine serum albumin (*w/v*) (BSA)) with 4 µL/mL liposomes with or without IRDye-700DX (200 µg dye/mL liposomes). After incubation at 37 °C for 24 h, cells were washed with binding buffer. Subsequently, cells were irradiated with a NIR light-emitting diode (LED) [20] (peak emission wavelength 690 nm (±10 nm for minimum and maximum emission), forward voltage: 2.6 V, power output: 490 mW) using 126 individual LED bulbs to ensure homogenous illumination at different radiant exposures (between 0 and 50 J/cm^2^) (LEDfactory, Leeuwarden, The Netherlands). All experiments were carried out in triplicate. The LED device was calibrated using a FieldMaxII-TO power meter with a PM2 sensor (thermopile head), resolution 1 mW (Coherent, Richmond, CA, USA).

Cell viability was measured 4 h after irradiation. During this time, the cells were kept at 37 °C and 5% CO_2_. To determine the viability, ATP content was measured using a CellTiter-Glo^®^ luminescent assay (Promega Benelux, Leiden, The Netherlands) according to the instructions of the manufacturer. Luminescence was measured using a TECAN infinite M200 Pro plate reader (PerkinElmer, Groningen, The Netherlands). The ATP content as a measure of cell viability was expressed as a percentage, determined by comparing the luminescent signal with the signal from untreated cells, which were considered 100% viable.

### 2.4. Collagen-Induced Arthritis

CIA was induced in male DBA/1JRj mice as described previously [21]. Briefly, animals received two injections of 100 µg bovine collagen type II (Radboudumc in-house production, batch 03-04-08). The first dose was emulsified in complete Freund’s adjuvant (FCA) and injected intradermally at the base of the tail. The second dose of FCA was administered in PBS intraperitoneally at day 21, with subsequent arthritis developed between days 21 and 25. Mice were scored 3 times a week for development of arthritis on each paw according to a 2-point scale of swelling and redness, as described previously [22]. Mice with a score ≥0.5 in one of the hind paws were included in the study. A cumulative inflammation score ≥7 was considered a humane endpoint.

### 2.5. Biodistribution

Male DBA/1JRj mice (starting weight 24.3 ± 0.8 g (*n* = 10/group)) with CIA were randomly divided between the treatment groups upon development of overt arthritis (macroscopic score of inflammation > 0.5). Upon inclusion, the mice were injected intravenously with 5 µL liposomes (in 200 µL PBS) with or without 1 µg IRDye700DX and labeled with 0.6 MBq ^111^In for biodistribution analysis. A subset of mice (*n* = 2 per group) were injected with 18 MBq ^111^In for SPECT/CT imaging. After 24 h, the mice were sacrificed, and the relevant tissues were dissected and weighed. Tissue uptake of ^111^In was determined using a γ counter (WIZARD, 2480 Automatic Gamma Counter, Perkin Elmer, Waltham, MA, USA). Results are depicted as percentage of the injected amount per gram tissue (%IA/g). Mice which received a SPECT/CT dose of ^111^In were scanned for 60 min (4 × 15 min frames) using a 1-mm-diameter pinhole ultra-high sensitivity mouse collimator (U-SPECT/CT-II, MILabs, Houten, The Netherlands). SPECT scans were followed by CT scans (65 kV, 615 µA). The SPECT scans were reconstructed using software from MILabs using a 0.2 mm voxel size, 1 iteration and 16 subsets.

### 2.6. Microscopic Scoring of Inflammation

To assess the joint inflammation, microscopically ankle joints were formalin-fixed, decalcified using formic acid and paraffin-embedded. The joints were subsequently cut into 7 µm sections, hematoxylin- and eosin-stained, and scored for the presence of inflammation according to the SMASH recommendations [23].

### 2.7. In Vivo Photodynamic Therapy

Male DBA/1JRj mice (starting weight 23.4 ± 1.4 g, (*n* = 8/group)) with CIA were randomly divided between the treatment groups upon development of overt arthritis in one of the hind paws (arbitrary arthritis score of >0.5). Mice were injected intravenously with 5 µL liposomes with or without 1 µg IRDye700DX in 200 μL of PBS, and 24 h after injection, the hind legs of the animals were exposed to 8.8 J/cm^2^ or 26.4 J/cm^2^ 690 nm light. The mice receiving the control liposomes also received the high dose of light. Arthritis development was subsequently scored daily. Mice were sacrificed 5 days after treatment, and the front and hind paws were formalin-fixed, decalcified using formic acid and paraffin-embedded. Histological sections of 7 µm were stained using hematoxylin and eosin, or safranin O, and scored for inflammation and proteoglycan depletion (PG depletion), respectively.

### 2.8. Statistical Analysis

Results are presented as mean ± SD. Statistical significance was determined using GraphPad Prism software (Version 5.03; GraphPad Software, San Diego, CA, USA). The tests used were: two-way ANOVA with Bonferroni post-test for the in vitro PDT, biodistribution, and therapy experiment. In the latter, we also accounted for repeated measures. The correlation between arthritis and liposome uptake in inflamed paws was determined using linear regression. A *p*-value < 0.05 was considered significant.

## 3. Results

### 3.1. Fluorescence and Absorption of IRDye700DX-Loaded Liposomes Is Similar to Free IRDye700DX

The fluorescence emission spectrum and absorbance spectrum were measured for liposomes with and without IRDye700DX loading, and these were compared to the respective spectra for free IRDye700DX. Results were normalized to peak fluorescence and absorbance for the corresponding measurement. The absorbance spectrum of 700DX-liposomes was very similar to that of free IRDye700DX, with both showing peak absorbance at 690 nm (Figure S1A). The fluorescence emission spectra were also very similar, with only a very minor shift in peak emission observed (Figure S1B).

### 3.2. PDT Using 700DX-Liposomes Induces Cell Death in a Light Dose Dependent Manner

**Figure 1:**
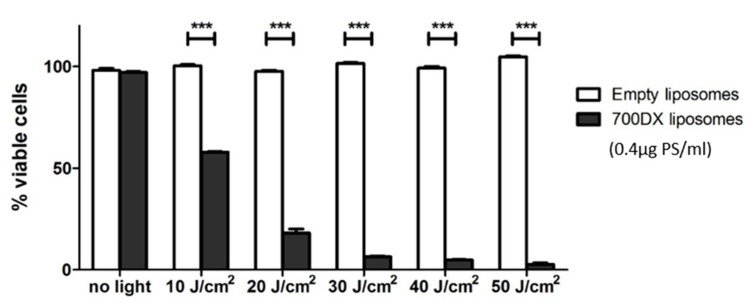
PDT using 700DX-liposomes in RAW 264.7 cells. Cell viability decreased in a light dose dependent manner after incubation with IRDye700DX-liposomes. Cell viability was not affected in cells incubated with control liposomes for any of the tested light doses. Data were normalized to the luminescent values of cells not incubated with liposomes and not exposed to light, and are depicted as mean ±SD (*n* = 3). Two-way ANOVA with Bonferroni post-test, *** = *p* < 0.001.

Already at light doses as low as 10 J/cm^2^ radiant exposure of 690 nm light, in vitro cell viability of cells incubated with 700DX-liposomes was approximately 50% compared to the untreated control (Figure 1). By increasing the light dose even higher, cell death could be generated until, at a light dose of 50 J/cm^2^, only 3% of cells remained. Radiant exposure after incubation with control liposomes without IRDye700DX did not cause cell death, nor did incubation with 700DX-liposomes without light exposure (both *p* > 0.5).

### 3.3. Liposomal Accumulation in the Arthritic Joint Is Not Negatively Affected by Loading with IRDye700DX

**Figure 2:**
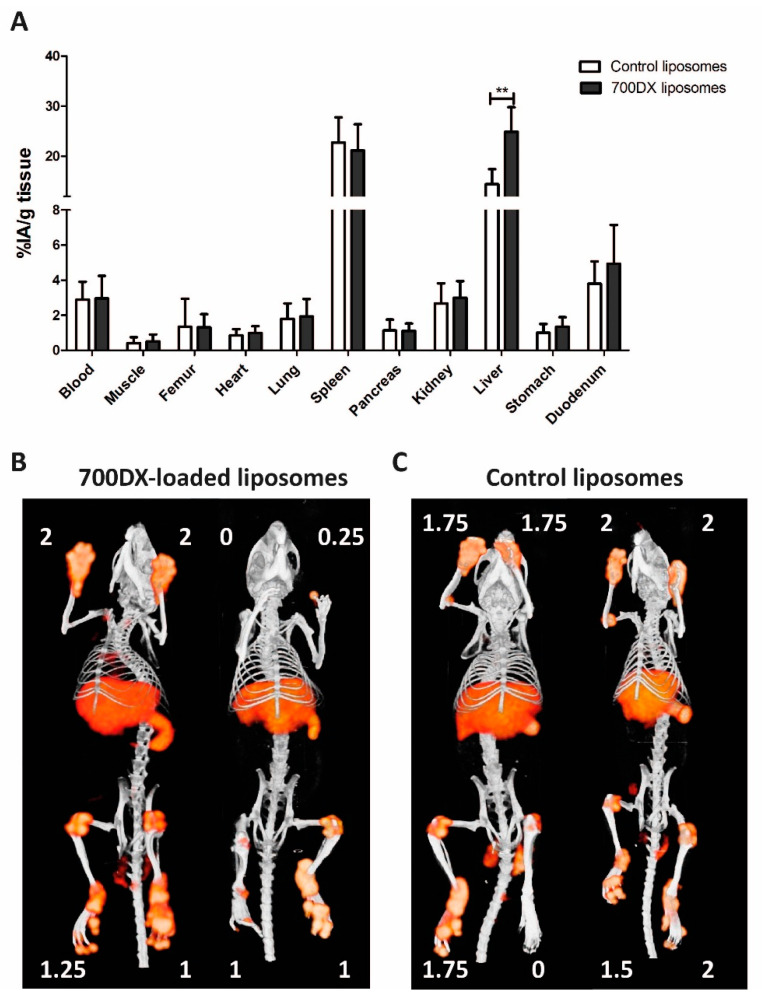
(**A**) Biodistribution of liposomes with or without IRDye700DX (*n* = 10 mice/group); (**B**) SPECT images of mice after injection of 111In-labeled IRDye700DX-liposomes; (**C**) SPECT images of mice after injection of ^111^In-labeled control liposomes. Arthritis scores are depicted in white next to the respective joints (** = *p* < 0.01).

**Figure 3:**
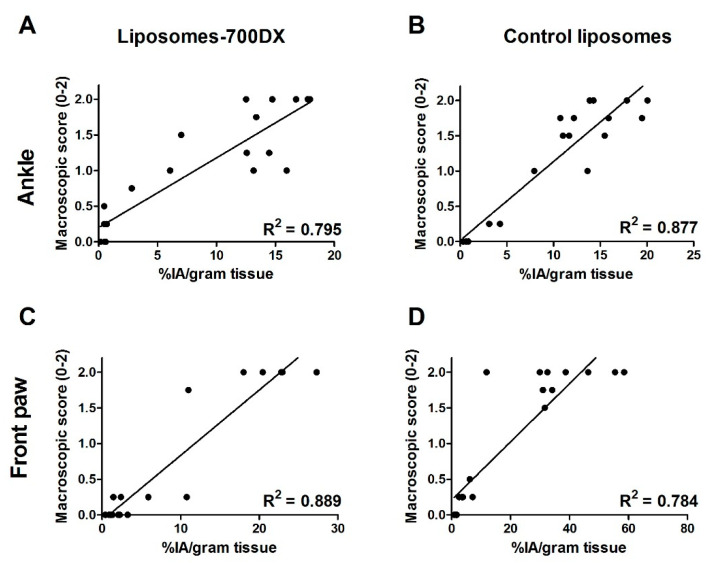
Uptake of 700DX-loaded and control liposomes positively correlated with macroscopic arthritis score in the hind ankle (**A**,**B**) and front paw (**C**,**D**) of mice with CIA (*n* = 10 mice).

The biodistribution of 111-indium-radiolabeled 700DX-liposomes in mice with CIA was comparable to that of the control liposomes (Figure 2). No significant difference in uptake was noted, with the exception of the liver, where accumulation of the liposomes with the photosensitizer was significantly increased (24.9 ± 4.9%IA/g versus 14.5 ± 3%IA/g for the liposomes with and without the PS, respectively (*p* < 0.01)). Tracer uptake by the inflamed regions correlated significantly for both constructs with the macroscopic score of arthritis both in the front paw (Figure 3a,b) and ankle joint (Figure 3c,d), as well as with the microscopic score of inflammation (Figure S2). SPECT/CT images that were acquired reflect these results, with clear uptake of the tracer observed in the inflamed joint, and with higher macroscopic inflammation scores also showing more uptake in the scans (Figure 2b,c).

### 3.4. PDT Using 700DX-Loaded Liposomes Ameliorates Arthritis Progression

**Figure 4:**
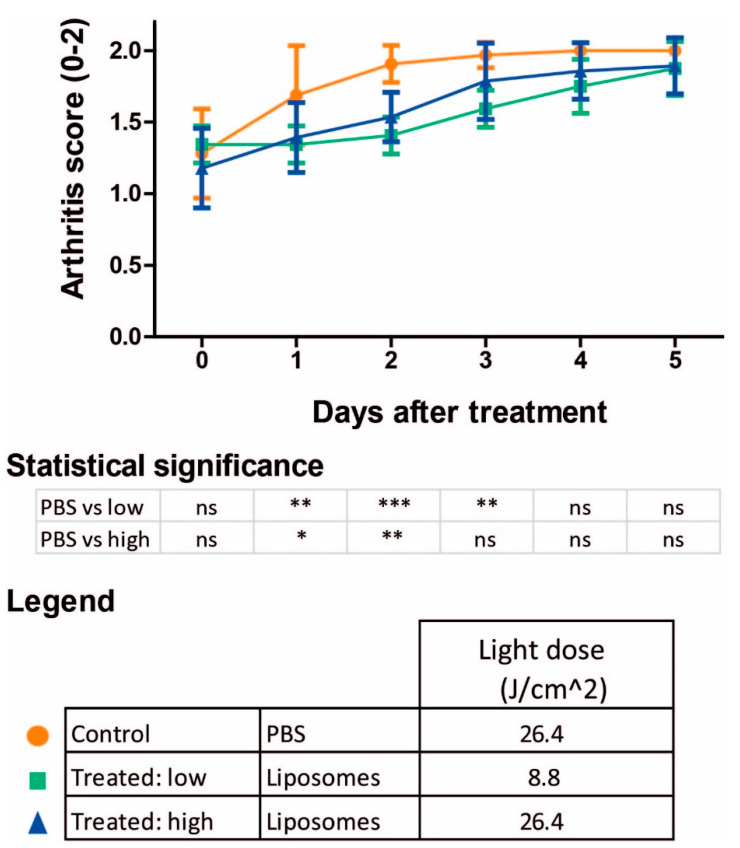
Development of arthritis over time after 700DX-liposome PDT. Initial arthritis development was slower after tPDT. Two-way ANOVA with Bonferroni post-test, * = *p* < 0.05, ** = *p* < 0.01, *** = *p* < 0.001, ns: not significant (*n* = 8 mice/group).

The therapeutic effect of PDT using 700DX-liposomes was investigated in male DBA/1JRj mice with CIA. The mice were injected intravenously with the 700DX-liposomes or PBS as a control, and the inflamed paws were exposed to the 690 nm light 24 h post-injection. The arthritis scores for both the 8.8 and 26.4 J/cm^2^ PDT treated groups were significantly lower than those for the PBS control at days 1–3 for the 8.8 J/cm^2^ and day 1 and 2 for the 26.4 J/cm^2^ PDT treated group (Figure 4). This difference became especially clear when accounting for the arthritis development of these mice as a whole using the area under the curve (Figure S3). After this initial delay in arthritis development in the treated paws, no differences in arthritis scores were observed at the later timepoints. In line with this, no differences in histological scores of inflammation or cartilage damage were observed after sacrifice of the animals at day 5 post-therapy (Figure S4).

## 4. Discussion

In this study, we demonstrated the feasibility of depleting macrophages using PS-loaded liposomes for tPDT both in vitro and in vivo in the CIA model of arthritis. Using PS-loaded liposomes, the macrophage cell line RAW264.7 was able to be efficiently depleted in a light dose dependent manner. The construct showed targeting of the arthritic joints in the CIA model of arthritis, with uptake closely correlating with arthritis severity in these mice. Finally, 700DX-liposome PDT was able to ameliorate arthritis development in the initial phase after treatment.

Macrophages are of critical importance for the development of RA. Depletion of macrophages using clodronate-loaded liposomes has previously been shown to be able to decrease the development of arthritis in mice when administered prior to overt arthritis development [12,13]. Additionally, arthritic flares were prevented, and administering the liposomes in the chronic phase of CIA ameliorated the development of the disease [24]. A drawback of the systemic action of clodronate-loaded liposomes is the resulting depletion of macrophages not only in the arthritic joints, but in many tissues, which interferes with the macrophage’s normal function of maintaining tissue homeostasis and preventing infection. By using a construct that is inert in the absence of light, we can deplete macrophages specifically in the arthritic joints, eliminating this problem.

Using PDT to treat arthritis in animal models has successfully been employed by several groups. Since these previous studies all used PDT, in which the PS preferentially accumulates in the arthritic joint due to the increased permeability of the vasculature, treatment was accompanied by significant side effects, because accumulation of the photosensitizer in neighboring skin and muscle tissue, which were inadvertently also exposed to light, could not be sufficiently prevented by untargeted approaches, and this resulted in necrosis in the surrounding musculature [25]. By using liposomes to deliver the photosensitizer preferentially to the macrophages, and thus increasing the uptake in the inflamed joints relative to neighboring tissues, these unwanted side-effects were successfully prevented in this study [9].

Current treatment for RA consists of systemic immunosuppression through the administration of biologic or synthetic DMARDS. The advantage of using 700DX-liposome PDT instead of, or complimentary to DMARDS is that the side effects of systemic immunosuppression, such as an increased risk of infectious diseases, can be avoided [26,27]. Furthermore, since 700DX-liposomes are systemically delivered, in contrast to other local adjuvant treatments such as chemical-, surgical- or radio-synovectomy, the inflamed joint is not damaged by the insertion of needles or other instruments.

An important hurdle to achieving clinical translation is the limitation of light penetration. Near infrared light has a penetration depth in tissue between 5 and 10 mm, which is suitable for the treatment of small joints, e.g., interphalangeal joints [28,29]. However, for larger joints, the light will not be able to penetrate deep enough to excite the PS in the whole joint. An alternative to reach the whole synovium could be to deliver the light directly into the joint using endoscopic or fibre-optic systems (reviewed in [28]).

In this study, loading of the liposomes with the photosensitizer IRDye700DX did not significantly change the biodistribution of the compound, with the exception of increased liver accumulation, where the compound is cleared by sinusoidal epithelial cells [30]. This effect has previously been described for other fluorophores and may be due to changes in the charge and lipophilicity of the compounds [31,32]. The time required for elimination of this construct was not determined in this experiment, since biodistribution analysis was only performed 24 h after injection. However, since the liver and spleen are not exposed to light, and the PS has no dark toxicity at the concentrations used in this model, this should not result in any off-target damage.

The limited and transient treatment effect observed in this study may have several explanations. First, using CIA as a model for experimental arthritis to study the effect of PDT using 700DX-loaded liposomes proved challenging in this study. Timing of the treatment and analysis of outcome measures were complicated by the highly variable disease course and incidence, and, since animals were included when they reached inclusion criteria, inclusion was spread over multiple weeks. At inclusion, the cumulative arthritis scores of the animals were variable, and we cannot rule out that the accompanying variation of systemic pro-inflammatory molecules influenced our treatment efficacy. Despite this limitation, we were able to demonstrate an amelioration of arthritis development. The transient nature of our therapy may also indicate that the treatment should be further optimized to give the best possible effect. Further optimization of the therapy could be achieved by performing light dosimetry, as well as by focusing on the timing of administration of the liposomes and subsequent light exposure [28,33]. Combining this therapy with low dose immunosuppressant therapy may also improve the longevity of the PDT effect. This is an especially relevant option for clinical translation since it allows for local treatment of disease flares without increasing systemic immunosuppression.

## 5. Conclusions

In conclusion, we have shown the proof-of-principle of performing photodynamic therapy in arthritic joints using liposomes loaded with a photosensitizer, which allows for locoregional treatment of RA and thus avoids systemic side effects. To expand on these findings and to assess their therapeutic value with respect to future clinical application, studies into PDT dosimetry and treatment effects in other animal models, as well as RA patient material ex vivo, should be performed.

## Data Availability

Data can be made available upon reasonable request.

## Supplementary Materials

The following are available online at https://www.mdpi.com/article/10.3390/pharmaceutics13111868/s1, Figure S1: Absorbance and fluorescence spectra of liposome with and without IRDye700DX compared to free IRDye700DX. Both absorbance and fluorescence spectra are similar when comparing IRDye700DX loaded in liposomes or free PS (all in PBS). Fluorescence emission was measured after excitation at 650 nm, Figure S2: Uptake of liposomes with (**a**) and without (**b**) IRDye700DX correlates with histological scores of inflammation in the ankle joints of mice with CIA (*n* = 10 mice), Figure S3: Area under the curve for the development of arthritis over time after 700DX-loaded liposome PDT. The AUC was significantly lower in the treated groups compared to PBS. Two-way ANOVA with Bonferroni post-test, * = *p* < 0.05 (*n* = 8 mice/group), Figure S4: Histological scores of ankle and knee joint inflammation, proteoglycan depletion and knee chondrocyte death and cartilage erosion. Results are depicted as mean with SD (*n* = 8 mice, (*n* = 7 for the 90 s exposed liposomes group)).

## Abbreviations

%IA/g: percentage of the injected amount per gram tissue; CIA: collagen-induced arthritis; DMARDs: disease-modifying anti-rheumatic drugs; DPPC: dipalmitoyl phosphatidylcholine; FCA: complete Freund’s adjuvant; IL-: Interleukin; PDT: photodynamic therapy; PEG-(2000)-DSPE: PEG-(2000)-distearoyl phosphatidylethanolamine; PG depletion: proteoglycan depletion; PS: photosensitizer; RA: rheumatoid arthritis; ROS: reactive oxygen species; SD: standard deviation; SPECT/CT: single photon emission computer tomography/computer tomography; TNF- α: tumor necrosis factor-α.
