# Supplementary material for: Photodynamic Therapy Targeting Macrophages Using IRDye700DX-Liposomes Decreases Experimental Arthritis Development
Source: Pharmaceutics. 2021 Nov 5;13(11):1868. doi: 10.3390/pharmaceutics13111868 (PMC8621465; doi:10.3390/pharmaceutics13111868)
Supplement: Supplementary file 1 [file pharmaceutics-13-01868-s001.zip › pharmaceutics-1400881-supplementary.pdf]

pharmaceutics

Supplementary Materials: Photodynamic Therapy Targeting Macrophages Using IRDye700DX-Liposomes Decreases Experimental Arthritis Development

Daphne N. Dorst, Marti Boss, Mark Rijpkema, Birgitte Walgreen, Monique M.A. Helsen, Desirée L. Bos, Louis van Bloois, Gerrit Storm, Maarten Brom, Peter Laverman, Peter M. van der Kraan, Mijke Buitinga, Marije I. Koenders and Martin Gotthardt

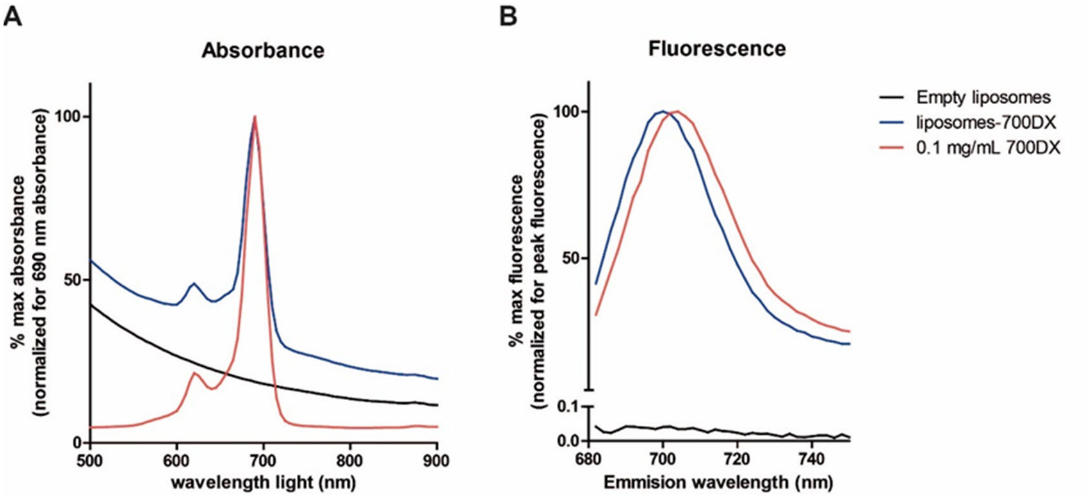

Figure S1. Absorbance (**A**) and fluorescence (**B**) spectra of liposome with and without IRDye700DX compared to free IRDye700DX. Both absorbance and fluorescence spectra are similar when comparing IRDye700DX loaded in liposomes or free PS (all in PBS). Fluorescence emission was measured after exci-tation at 650 nm.

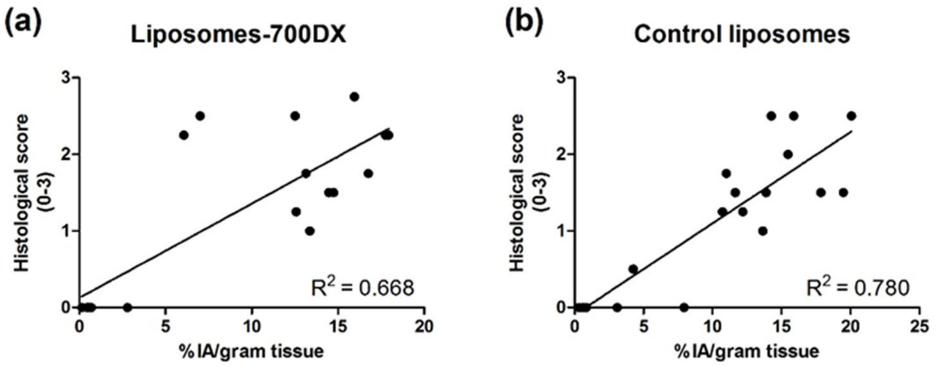

Figure S2. Uptake of liposomes with (**a**) and without (**b**) IRDye700DX correlates with histological scores of inflammation in the ankle joints of mice with CIA. (n=10 mice).

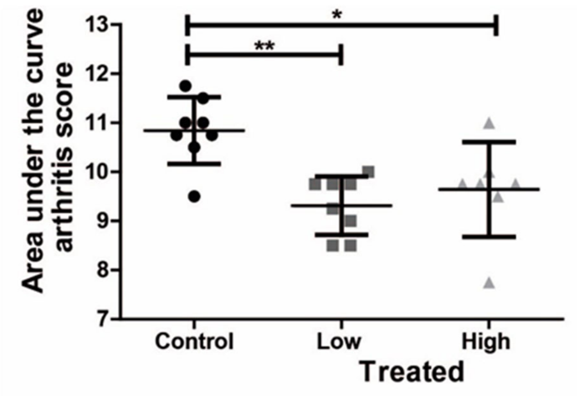

Legend

		Light dose (J/cm^2)
Control	PBS	26.4
Treated: low	Liposomes	8.8
Treated: high	Liposomes	26.4

Figure S3. Area under the curve for the development of arthritis over time after 700DX-loaded liposome PDT. The AUC is significantly lower in the treated groups compared to PBS. Two-way ANOVA with Bonferroni post-test, * = p <0.05, ** = p < 0.01 (n=8 mice/group).

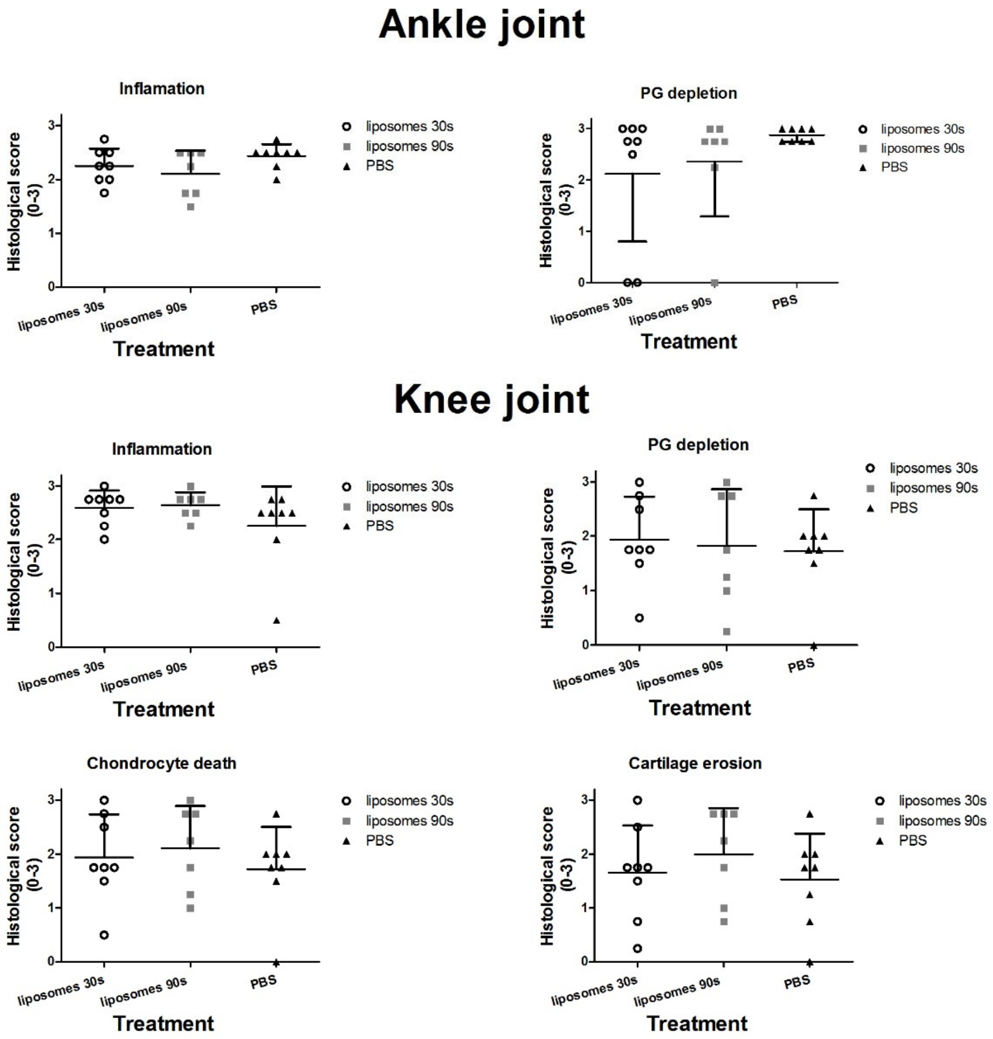

Figure S4. Histological scores of ankle and knee joint inflammation, proteoglycan depletion and knee chondrocyte death and cartilage erosion. Results are depicted as mean with SD (n = 8 mice, (n = 7 for the 90s exposed liposomes group)).
